# Medical Opinion and Movement

**Published:** 1910-06-04

**Authors:** 


### Medical Opinion and Movement.

### Vomiting of Pregnancy.

At a meeting of the *Académie de Médecine* Dr. Pinard opened a discussion on the vomiting of pregnancy. After referring to the dangers of obstinate and intractable vomiting, he insisted on the importance of variations in the pulse and the primary necessity of watching the condition of the pulse in these cases. According to this author acceleration of the pulse is the first manifestation of a condition of toxæmia, probably due to the secretion of a poison by the ovary or by the ovum. If, in spite of appropriate treatment—rest, milk diet, and inhalations of oxygen—the pulse reaches 100 and remains at this figure or exceeds it, in the absence of any elevation of temperature, the author is of opinion that this fact is in itself sufficient to justify putting an end to the pregnancy and bringing on abortion.

### Chloroform Narcosis and Infection.

In a contribution to *Morgagni* Dr. G. Zancarini gives an account of some observations carried out by him on animals and man to determine the effect of chloroform vapours upon the mucous membrane of the respiratory passages. He finds that chloroform narcosis produces a marked irritation and congestion of the nasal mucous membrane, which may even attain to an actual rhinitis. Microscopic examination of the nasal secretion shows an abundance of shed epithelium more or less degenerated. This active destruction of epithelium, which under normal circumstances offers resistance to the invasion and multiplication of microbes, renders conditions more favourable for the latter, and the author finds a notable increase in micro-organisms in the nasal fossæ in comparison with the condition previous to chloroformisation. By a series of researches the author finds further that in ordinary conditions pathogenic microbes are unable to reach the general system through the nasal mucous membrane, but after chloroform inhalation this barrier breaks down, and in animals in which some form of traumatism was produced under chloroform narcosis and certain microbes injected into the nose, these same microbes were found at the seat of the lesions. From these experiments the author draws the conclusion that post-operative infections in cases in which absolute aseptic precautions have been taken, may be due to the invasion of pathogenic micro-organisms through the irritated nasal mucous membrane. He suggests that chloroform should be administered as sparingly as possible so as to prevent irritation of the respiratory mucous membrane, and that special antiseptic precautions should be taken subsequently to prevent infection in this manner. If such are the risks with chloroform administration, they must be much intensified in ether narcosis, in which irritation of the respiratory passages is much more marked.

### The "Bacterium Anthroposepticum."

An interesting case is reported in the *Zeitschrift für Hygiene und Infektionskrankheiten* by Drs. Fränkel and Pielsticker from which they succeeded in isolating an entirely new pathogenic microbe. The case was that of a man, aged 33, who died with the clinical diagnosis of acute osteomyelitis of the left femur, and consequent septicæmia. Microscopic examination showed that it was not an ordinary osteomyelitis such as results from infection with the *staphylococcus pyogenes aureus*, the inflammatory process being entirely of a necrotic nature with formation of a purulent line of demarcation. Moreover, there were no metastases, and the whole condition suggested the presence of some pathogenic agent different from that usually found in such cases. Bacteriological examination of the diseased focus and of the blood fully confirmed this supposition. In cultures on blood numerous blackish-green colonies developed and grew rapidly. Microscopically the organism consisted of short ovoid rods with rounded ends more deeply coloured than the centre, resembling in appearance the plague bacillus. It was very mobile and measured about a third of the diameter of a human red blood-corpuscle. There were no spores. Cultures gave a well-marked peculiar aromatic odour. A large number of experimental inoculations were carried out to test the pathogenic properties of the microbe in relation to animals. With pigeons and fowls results were negative; but white mice succumbed rapidly. In guinea-pigs infraperitoneal inoculation produced acute purulent peritonitis with bacteriæmia, and death within twenty-four hours. Rabbits developed a typical pyæmia with metastatic abscesses in the internal organs, and a characteristic of these infections consisted in hæmorrhagic and purulent lesions of the testicle and epididymis. From this extensive research the authors conclude that this is a micro-organism hitherto undescribed and belonging to the bacterial group of hæmorrhagic septicæmia, and they propose to designate it under the name of *Bacterium Anthroposepticum*.

### Gastro-enterostomy.

Dr. A. Blad, of Copenhagen, has made some interesting observations to test the effect of the operation of gastro-enterostomy in regard to the digestive functions of the stomach. The observations were carried out upon twenty patients, on whom the operation was performed by Mr. Rosving for gastric ulcer. The gastric motricity and acidity were examined both before and after operation in the usual way by drawing off the stomach contents after a test meal. In twelve cases there was no marked difference in the way the stomach emptied itself before and after operation. In three of these there was no retention, and in nine only slight. In two cases the stomach appeared to empty itself better after operation, and in six cases there was increased retention after operation. The author suggests as causes for this increased retention, too great a reflux of bile and pancreatic juice owing to the anastomosis, or inhibition of gastric motricity from adhesions of the organ to the intestines formed around the anastomosis. Too much stress, however, must not be laid upon mechanical conditions, for even in those cases in which there was no change in this respect the general condition of the patient was much improved. This improvement, he thinks, may be ascribed to the neutralisation of excess of hydrochloric acid caused by the reflux of bile into the stomach, and he suggests that this reflux may also have a reflex inhibitory effect upon acid secretion. Observations on the gastric acidity generally showed a diminution after operation. Hyperacidity would therefore appear to be an especial indication for operation. On the other hand, gastric ulcer with gastric achylia and gastritis would contra-indicate operation. In fact, two cases of this nature in which Rosving operated showed no improvement.

### Trypsin in the Stools of Infants.

According to the experiments of Rovere, who reports them in the *Rivista di Clinica Pediatrica*, trypsin is not present in the meconium of new-born infants who have not been suckled, a fact which is explicable when it is remembered that intestinal peristalsis does not occur until it has been reflexiv excited by suckling. In the first year of life trypsin is found readily in the stools, and the function of the pancreas does not seem to be disturbed by intestinal derangements, for the author has discovered the ferment in health and in disease. He concludes that trypsin is present in the fæces of infants as early as the second or third day of life, and that it is never absent even in the most varied diseases.

### Absorption of CO after Death.

Continuing the investigations of Strassmann and Schulz, which were published some time ago, Stoil, in the *Vierteljahrschrifte f. Gericht. Med.* shows that there is no difficulty in diagnosing whether carbon monoxide gas has been inhaled during life or after death. His experiments made on the bodies of children were undertaken as the result of the doubts which existed, as to whether exposure of a body for a considerable time to the action of carbon monoxide gas could cause the blood to become so saturated with the gas, as to render the fact of the *intra-vitam* inhalation of the gas impossible of definite determination. He finds that, after testing the blood of the internal organs of bodies so exposed, by the tannin, acetic acid and quinine tests as well as by the spectroscope, the presence of carbon monoxide in the blood is a certain indication that the gas was absorbed during life.

### Beri-beri.

Fraser and Stanton, who have recently been working at the relationship of beri-beri to rice in the Federated Malay States, have now reached some very important conclusions. They believe that beri-beri is a disorder of nutrition, and, as it occurs in the Malay Peninsula, is associated with a diet in which white rice is the principal constituent. The white rice as produced by the mills in Malaya commonly makes default in respect of some substance or substances essential for the maintenance of the normal nutrition of nervous tissues; these substances exist in adequate amount in the original grain and in superabundant amount in the polishings from white rice. The estimation in terms of phosphorus pentoxide of the total phosphorus present in a given rice may be used as an indicator of the beri-beri producing power of such rice when forming the staple of a diet in man. The authors believe that the prevention of beri-beri in the Federated Malay States will be achieved by substituting for the ordinary white rice a rice in which the polishing process has been omitted or carried out to a minimal extent, or by the addition to a white rice diet of articles rich in those substances in which such white rice now makes default. Polishing, from white rice or the parboiled (cured) rice of Braddon will meet this end, the former article having the advantage of being cheap and of being readily obtained. These results are of great importance to the Malay States and other places in the East, and they must of a necessity more or less completely revolutionise the milling of rice as it is at present carried out in these places.

### Widal Reaction for Typhoid in Tuberculosis.

Recent investigations seem to show a possible source of fallacy in that tuberculous subjects free from any suspicion of typhoid or paratyphoid infection may yet give the Widal agglutination reaction. At a meeting of the Royal Academy of Medicine of Belgium on October 30, 1909, Hermann described experiments with twelve rabbits which before inoculation with tubercle gave no Widal reaction, the dilution employed being 1 in 50. Two months after infection with tubercle all the twelve gave a clear macroscopic reaction with a dilution of 1 in 40, and when killed and examined they all showed more or less advanced tuberculous lesions. Further, the same author obtained a positive result with a dilution of 1 in 50 in two out of three cases of advanced consumption in the human subject. He suggests that the dilution commonly used in performing this test is therefore not high enough to exclude fallacy. Krenecker also (*Münch. Med. Woch.*, No. 20, 1909) says that a positive Widal test can be obtained in pyrexial tuberculosis, and may again disappear in the course of the disease when there is no typhoid infection. In view of the frequency of some tuberculous focus, these statements seem worthy of note.

### Treatment of Spasmodic Asthma.

The virtues of adrenalin injected hypodermically for the relief of spasmodic asthma have long been known, but not so widely as might be thought when the tendency of asthmatics to try one drug after another is kept in mind. Dr. Melland, writing in the *Lancet*, not only quotes three cases from his own practice in which this remedy was efficacious when all others failed, but also enters fully into a theoretical explanation based upon the physiological actions of the drug. As a matter of practical therapeutics this application of adrenalin solution is probably more practised than he appears to think; but it is quite true that it is less generally known than it deserves to be. The usual dose for this purpose is five minims, and it gives the most striking relief almost instantly if it is going to act at all; given by the mouth there is practically no effect, even when much larger doses are used. It is in the work of Dr. Gaskell especially that Dr. Melland finds what he believes to be the explanation of this power which adrenalin has to relax the spasm of the bronchioles. All the involuntary muscles supplied by the thoracico-lumbar outflow are contracted by adrenalin, which causes the same results as stimulation of the nerves themselves. This action is confined entirely to the nerves of this sympathetic system, and never resembles that of stimulation of either the cranial or sacral visceral nerves. Dr. Melland goes further than Dr. Gaskell, for he asserts that adrenalin actually inhibits all involuntary muscles supplied by the cranial and sacral outflows. He explains the well-known action of violent emotion in arresting a paroxysm of asthma in the same way; that is, by supposing that the stimulation of the sympathetic inhibits the vagus, and so brings about the same result as the injection of adrenalin.

### Contracted Pelvis.

The widely diverse opinions and teachings still prevalent concerning the best methods of conducting labour when there is contraction of the pelvis were illustrated at a recent meeting of the Obstetrical Section of the Royal Society of Medicine, when Dr. Hastings Tweedy read a paper on this subject. Practically all the English obstetricians disagreed strongly with some of the principles propounded, especially on the question of induction of premature labour. Dr. Tweedy recognises for practical purposes five degrees of contracted pelvis. The most extreme contains all pelves of 2¼ inches (or less) in the conjugate; generally contracted pelves are treated as worse by half an inch than those with simple flattening. The other four degrees are separated by half-inch measurements. Since an abnormally large child can be safely delivered through a normal pelvis uninjured, a full-sized fœtus may also pass through a contracted pelvis of the first and second degree. In such pelves normal delivery is to be anticipated, and interference is warranted only when complications arise. For such cases prophylactic turnings and induction of premature labour are never indicated. Indeed, he says the latter is never advisable, though version is admissible for such complications as prolapse of the cord with contraction of the first or second degree, but not for contraction alone. In the third degree of contraction time should not be wasted in an endeavour to obtain natural delivery; Cæsarean section is the operation of choice, provided the patient is seen early in labour and the child is alive; otherwise subcutaneous pubiotomy or symphysiotomy (which are about of equal value) should be performed. In the fourth and fifth degrees extra peritoneal Cæsarean section is the operation of choice when the classical Cæsarean section is contraindicated. High forceps should never be applied until preparations are complete for enlarging the pelvis, when they may be tried tentatively. These views were very strongly combated during the subsequent discussion.

### Pubiotomy for Dystocia.

Notwithstanding the disfavour with which most obstetricians in this country regard pubiotomy, the subcutaneous operation seems to be gaining favour elsewhere. Dr. Whitridge Williams, in the *American Journal of Obstetrics and Gynæcology*, considers the subject exhaustively on the strength of twenty-five cases. He had no maternal and three fœtal deaths; but ascribes one only of the latter to the operation. All patients were delivered by forceps or version immediately after the pubiotomy; there were no injuries to the bladder, but six perineal and five deep vaginal tears took place—not a great number since twelve of the patients were primiparæ. The operation is restricted to border-line cases, is not done until a prolonged second stage has shown that natural delivery will not take place, and is done only in clean cases and by Döderlein's technique. The after results are satisfactory; there is definite mobility between the ends of the bone in two-thirds of the patients, but this seldom interferes with full bodily activity. The indications for subcutaneous pubiotomy are a conjugata vera of not less than 7 cm. (2⅘ inches) and a sufficient test of the second stage of labour. In multiparæ with a history of repeated dystocia, or in primiparæ with excessive contraction, pubiotomy is inferior to Cæsarean section at the onset of labour; but the two do not really enter into competition, for when labour has already been a long time in progress pubiotomy is many times the less dangerous of the two. In uninfected women it should replace high forceps, prophylactic version, induction of premature labour, and craniotomy of the living child. In some cases permanent enlargement of the pelvis follows the operation, and subsequent labours may terminate spontaneously; but the operation can be done again if necessary.

### The Bacillus of Whooping-Cough.

As the result of experiments on monkeys and young dogs Klimenko, who publishes his experiments in the *Centralbl. f. Bakl. u. Parasiten*, concludes that the Bordet-Gengon bacillus is certainly the cause of whooping-cough. The injection of pure cultures of this organism will cause the disease in the animals mentioned above, and in all probability in other animals also. The bacillus can be recognised culturally in all recent cases in the expectoration. He was unable to produce infection in rabbits, mice, lambs, guinea-pigs, and sucking-pigs. Older dogs showed more resistance to the infection than young ones. In these last, and in monkeys, the resemblance was striking between the symptoms of the experimentally-provoked disease and those of the human whooping-cough, extending as it did even to the wheezy-cough. A minute account is given of the morphological and cultural characteristics of the organism.

